# Underweight is a major risk factor for atrial fibrillation in Asian people with type 2 diabetes mellitus

**DOI:** 10.1186/s12933-021-01415-2

**Published:** 2021-11-24

**Authors:** Jung-Chi Hsu, Yen-Yun Yang, Shu-Lin Chuang, Yi-Wei Chung, Chih-Hsien Wang, Lian-Yu Lin

**Affiliations:** 1 Division of Cardiology, Department of Internal Medicine, Fu Jen Catholic University Hospital, Fu Jen Catholic University, New Taipei City, Taiwan; 2 Division of Cardiology, Department of Internal Medicine, National Taiwan University College of Medicine and Hospital, Taipei, Taiwan; 3 Graduate Institute of Epidemiology and Preventive Medicine, College of Public Health, National Taiwan University, Taipei, Taiwan; 4 Department of Medical Research, National Taiwan University Hospital, Taipei, Taiwan; 5 Division of Cardiology, Department of Internal Medicine, Shuang Ho Hospital, Taipei Medical University, New Taipei City, Taiwan; 6 Taipei Heart Institute, Taipei Medical University, Taipei, Taiwan; 7 Cardiovascular Research Center, Taipei Medical University Hospital, Taipei, Taiwan; 8 Division of Cardiovascular Surgery, Department of Surgery, National Taiwan University College of Medicine and Hospital, Taipei, Taiwan; 9 Cardiovascular Center, National Taiwan University Hospital, Taipei, Taiwan

**Keywords:** Body mass index, Atrial fibrillation, Obesity paradox, Diabetes mellitus

## Abstract

**Background:**

Atrial fibrillation (AF) is prevalent in patients with type 2 diabetes mellitus (T2DM). Obesity commonly accompanies T2DM, and increases the risk of AF. However, the dose-relationship between body mass index (BMI) and AF risk has seldom been studied in patients with diabetes.

**Methods:**

This cohort study utilized a database from National Taiwan University Hospital, a tertiary medical center in Taiwan. Between 2014 and 2019, 64,339 adult patients with T2DM were enrolled for analysis. BMI was measured and categorized as underweight (BMI < 18.5), normal (18.5 ≤ BMI < 24), overweight (24 ≤ BMI < 27), obesity class 1 (27 ≤ BMI < 30), obesity class 2 (30 ≤ BMI < 35), or obesity class 3 (BMI ≥ 35). Multivariate Cox regression and spline regression models were employed to estimate the relationship between BMI and the risk of AF in patients with T2DM.

**Results:**

The incidence of AF was 1.97 per 1000 person-years (median follow-up, 70.7 months). In multivariate Cox regression, using normal BMI as the reference group, underweight (HR 1.52, 95% CI 1.25–1.87, *p* < 0.001) was associated with a significantly higher risk of AF, while overweight was associated with significantly reduced risk of AF (HR 0.82, 95% CI 0.73–0.89, *p* < 0.001). Kaplan–Meier analysis showed AF risk was highest in the underweight group, followed by obesity class 3, while the overweight group had the lowest incidence of AF (log-rank test, *p* < 0.001). The cubic restrictive spline model revealed a “J-shaped” or “L-shaped” relationship between BMI and AF risk.

**Conclusions:**

Underweight status confers the highest AF risk in Asian patients with T2DM.

**Supplementary Information:**

The online version contains supplementary material available at 10.1186/s12933-021-01415-2.

## Introduction

Type 2 diabetes mellitus (T2DM) and atrial fibrillation (AF) are global public health challenges and major causes of death and cardiovascular events [1]. A multitude of studies have indicated DM is an independent risk factor for AF, in conjunction with a coexisting precipitating environment for AF [2, 3]. The trends in obesity—one of the most well-characterized, notorious risk factors—closely mirror the trends in the prevalence of T2DM. In the United States, 61% to 85% of people with T2DM are overweight or obese [4, 5]. Moreover, obesity is also an established risk factor for AF. Compared to nonobese individuals, obesity increases the risk of developing AF by 49% in the general population, and the risk escalates in parallel with body mass index (BMI) [6, 7]. In the Framingham Heart Study, every unit increase in BMI correlated with a 4–5% increase in AF risk [8]. Moreover, a population-based study showed that each 1 and 5 kg/m^2^ reduction in BMI were associated with a 7% and 12% reduction in the risk of new-onset AF [9].

BMI is the most commonly used parameter to determine the degree of obesity. However, data on BMI and AF in T2DM populations are relatively scarce. Body weight and DM were reported to have a synergistic effect on the risk of new-onset AF [10]. Although DM is usually associated with overweight and obesity, its prevalence among normal-weight individuals is increasing. Concerningly, while Asian individuals are more likely to be overweight and less likely to be obese, they are 30–50% more likely to develop DM than their white counterparts, despite having a lower BMI [11]. However, significant racial and ethnic disparities in the definition of obese based on BMI cutoffs continue to persist. In a recent study, the prevalence of T2DM was 5.4% and 23.5% among underweight and normal-weight Asian men and 0.0% and 6.1% in their White male BMI counterparts. The prevalence of T2DM was found to be 5.6% and 13.6% in underweight and normal weight Asian women and 2.3% and 2.8% in underweight and normal weight White women [12].

The intriguing observations of J- and U-shaped distributions between BMI and cardiovascular complications and mortality imply that the impact of being underweight may be overlooked. In fact, evidence indicates that, among patients with AF, underweight individuals are a special population that need additional care. For example, among patients with AF taking direct oral anticoagulants (DOACs), being underweight was associated with an increased risk of major bleeding, thromboembolism, and death [13–15]. However, the risks associated with being underweight are rather difficult to address, especially as a relatively small proportion of Caucasians are underweight compared to Asians. There is a lack of compelling evidence on the obesity paradox and the risk of AF in patients with diabetes. Thus, this study aimed to explore the dose-relationship between BMI and AF risk in patients with T2DM.

## Methods

### Study population

We evaluated longitudinal data on Taiwanese patients diagnosed with T2DM aged 50 years or older at a tertiary medical center between January 1, 2014, and December 31, 2019. Detailed medical records were obtained from the well-established National Taiwan University Hospital integrated Medical Database (NTUH-iMD), which is based on the International Classification of Diseases, tenth revision codes and ATC (Anatomical Therapeutic Chemical Classification) drug codes, and regulated examination codes in Taiwan. The study was approved by the Institutional Review Board (IRB) of National Taiwan University Hospital.

Patients with previous AF before the onset of T2DM or who were lost to follow-up (defined as a lack of follow-up at the outpatient clinic for more than three months) were excluded. Baseline characteristics including hypertension (HTN), hyperlipidemia, gout, heart failure, coronary artery disease (CAD), valvular heart disease (VHD), chronic kidney disease (CKD), chronic obstructive pulmonary disease (COPD), and peripheral arterial occlusive disease (PAOD) were obtained from the patients’ electronic health records (EHRs). The ICD codes and the numbers of patients’ visits were summarized in Additional file 1: Tables S1 and S2. Estimated glomerular filtration rate (eGFR) was calculated by the modification of diet in renal disease (MDRD) equation. History of transient ischemic accident (TIA) or ischemic stroke was defined as the occurrence of TIA or ischemic stroke before the diagnosis of DM, and history of heart failure was defined as patients who had been hospitalized due to acute decompensated heart failure. Diagnoses of cancer were also recorded. Prescribed drugs were categorized as antiarrhythmic agents; calcium channel blockers (CCB); beta-blockers; angiotensin converting enzyme inhibitors (ACEI); angiotensin receptor blockers (ARB); mineralocorticoid-receptor antagonists (MRA); anticoagulants including direct oral anticoagulant (DOAC) and warfarin; and anti-diabetic medications including insulin, metformin, sodium-glucose co-transporter-2 (SGLT2) inhibitors, dipeptidyl peptidase 4 (DPP4) inhibitors, sulphonylurea, repaglinide, acarbose, thiazolidinedione (TZD), and glucagon-like peptide-1 (GLP-1) agonists. Echocardiographic studies were performed using a Phillips iE33 (Phillips, Bothell, WA, USA) and two‐dimensional‐guided M‐mode measurements with a 3.0‐ or 3.5‐MHz transducer. Left atrium (LA) size, left ventricular internal dimension in end‐diastole (LVIDd) and systole (LVIDs), and left ventricular ejection function (LVEF) were collected in the parasternal long-axis view with a M-mode cursor. LA size was measured as the anterior–posterior diameter in end-ventricular systolic phase. Left ventricular mass (LVM) was calculated using the Devereux formula. All echocardiographic data were obtained from the EHRs.

### Data measurement

Body height was measured using a stadiometer against the wall. Subjects stood in an upright position on the flat surface of the stadiometer without shoes, with the back of their heels and occiput on the stadiometer. Body height was recorded in centimeters (cm) and rounded off to the first decimal place. Body weight was measured using an electronic digital scale and recorded in kilograms (kg) to the nearest 0.1 kg. BMI was calculated by dividing weight in kilograms by height in meters squared (kg/m^2^). Subjects were categorized into five BMI groups following the recommendations from the Health Promotion Administration, Ministry of Health and Welfare, Taiwan: underweight, BMI < 18.5; normal range, 18.5 ≤ BMI < 24; overweight, 24 ≤ BMI < 27; obese class 1 (mild), 27 ≤ BMI < 30; obese class 2 (moderate), 30 ≤ BMI < 35; and obese class 3 (severe), BMI ≥ 35.

New-onset AF and its date of occurrence were identified based on the diagnosis code from either the EHRs or standard 12-lead electrocardiograms. The end points of this study were occurrence of new-onset AF, last clinical visit, or death.

### Statistical analysis

Continuous variables are described as means (SD) and categorical variables are presented as frequencies (percentages). Differences among groups were tested using the Chi square test for categorical variables and analysis of variance (one-way ANOVA) test for continuous variables. The relationship between BMI and AF was assessed using multivariate Cox’s regression models, from which hazard ratios (HRs) and 95% confidence intervals (CIs) were derived. We adjusted the confounders step-by-step to make sure the associations remained consistent through increasingly complex models. In the basic model (1), we adjusted for baseline characteristics including age, gender (using male as the reference group), hyperlipidemia, gout, history of heart failure, VHD, CAD, COPD, PAOD, prior TIA/ischemic stroke, baseline HbA1C, baseline FG, and baseline eGFR. Subsequently, we further adjusted model 2 using three echocardiogram parameters: baseline LA size, LVEF, and LVM. The estimated cumulative incidences for AF were derived using the Kaplan–Meier approach; the significance of the differences between curves were examined using the log-rank test.

As non-linear dose–response associations were expected, restricted cubic splines with five knots located at the 5th, 27.5th, 50th, 72.5th and 95th percentiles of the BMI distribution were used to determine the relationship between BMI and AF [16]. We also conducted subgroup analyses stratified by gender and age. Missing values were discarded. The Forest plots are displayed for subgroup analyses, with adjusted hazard ratios (aHR) along with confidence intervals and *p*-values plotted for each variable.

A two-tailed *p*-value of less than 0.05 was considered statistically significant. All statistical analyses were performed using R version 3.6.2 (University of Auckland, Auckland, New Zealand) and SPSS statistical software 25.0 (SPSS Inc., Chicago, IL, USA).

## Results

### Baseline characteristics

**Fig. 1:**
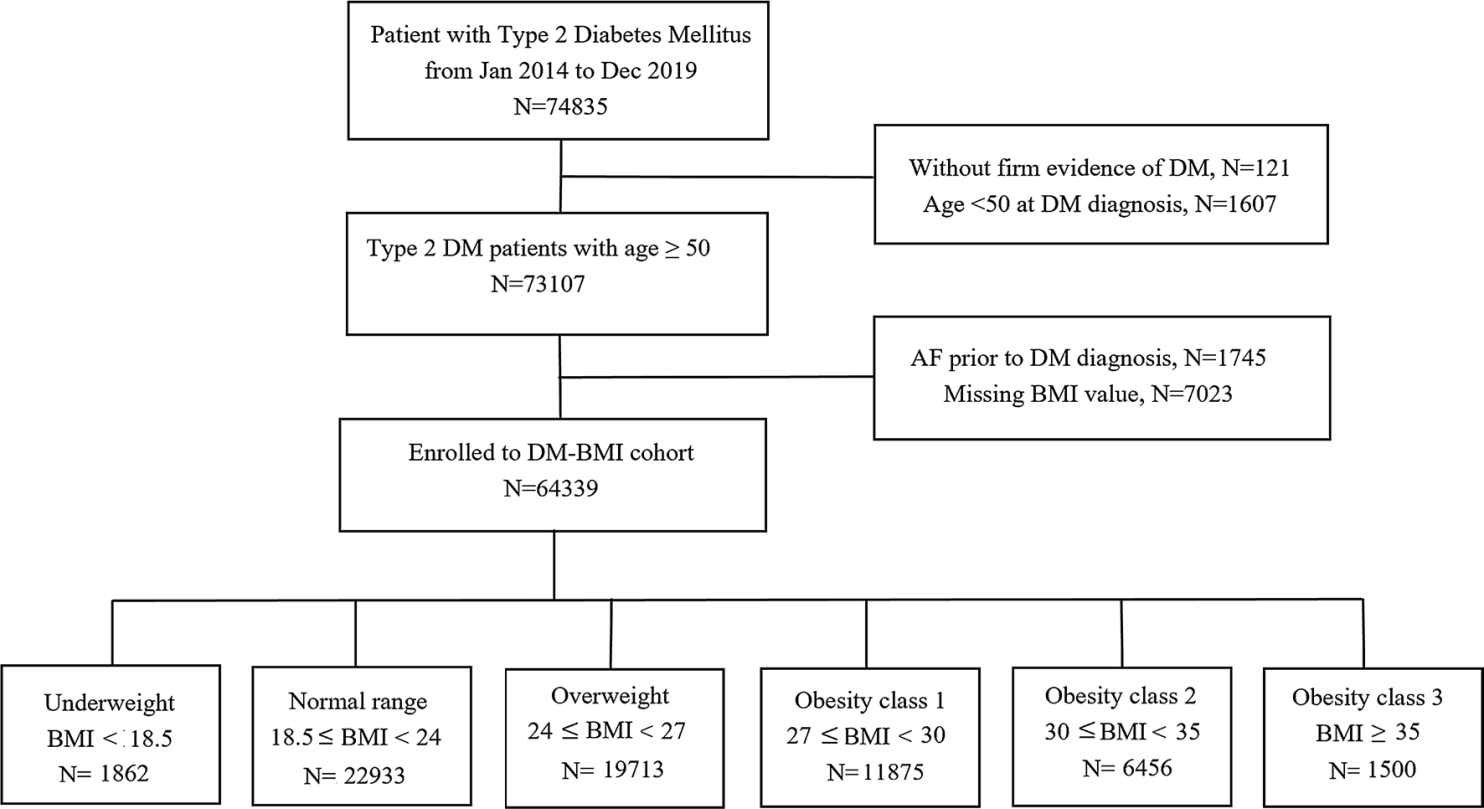
Study flowchart

The flowchart of patient selection is demonstrated in Fig. 1. A total of 74,835 patients with a T2DM diagnosis code between 2014 and 2019 were initially enrolled. Of these, 121 patients without firm evidence of T2DM (only one blood test or not prescribed DM medications) and 1607 patients aged below 50-years-old were excluded. We also excluded 1745 patients with pre-existing AF, and 7023 patients with missing BMI values. Finally, a total of 64,339 subjects were enrolled for the DM-BMI cohort analysis.

Among the 64,339 study subjects, 2.9% (*n* = 1862) were classified as underweight, 35.6% (*n* = 22,933) as normal BMI, 30.6% (*n* = 19,713) as overweight, 18.5% (*n* = 11,875) as obesity class 1, 10.0% (*n* = 6456) as obesity class 2, and 2.33% (*n* = 1500) as obesity class 3. The baseline characteristics are shown in Table 1. The underweight patients were older and had a poorer baseline eGFR, while the severely obese patients were more likely to have cardiovascular risk factors such as hypertension, hyperlipidemia, gout, and CKD. After a median follow-up period of 70.7 months, 5692 individuals (8.8% of the total population, 1.97 per 1000 person-years) developed AF. The incidence of AF was 3.60, 2.03, 1.86, 1.95, 1.88, and 2.25 per 1000 person-years for the underweight, normal range, overweight, obesity class 1, obesity class 2, and obesity class 3 groups, respectively.

**Table 1:** Baseline Characteristics According to BMI Categories

	Total *n* = 64,339	Underweight BMI < 18.5 *n* = 1862	Normal range 18.5 ≤ BMI < 24 *n* = 22,933	Overweight 24 ≤ BMI < 27 *n* = 19,713	Obesity class 1 27 ≤ BMI < 30 *n* = 11,875	Obesity class 2 30 ≤ BMI < 35 *n* = 6456	Obesity class 3 BMI ≥ 35 *n* = 1500	*P*-value
Age (years)	67.7 ± 9.9	71.6 ± 11.6	68.9 ± 10.1	67.6 ± 9.7	66.7 ± 9.6	65.1 ± 9.3	63.0 ± 8.6	< 0.001
< 65	26,723 (41.6)	559 (30.0)	8443 (36.8)	8112 (41.2)	5352 (45.1)	3360 (52.0)	897 (59.8)	
65–74	20,989 (32.6)	564 (30.3)	7596 (33.1)	6632 (33.6)	3826 (32.2)	1933 (29.9)	438 (29.2)	
≥ 75	16,627 (25.8)	739 (39.7)	6894 (30.1)	4969 (25.2)	2697 (22.7)	1163 (18.1)	165 (11.0)	
Male	33,930 (52.7)	813 (43.7)	11,486 (50.1)	11,241 (57.0)	6565 (55.2)	3199 (49.6)	626 (41.7)	< 0.001
Body height (cm)	160.8 ± 8.5	159.8 ± 8.3	160.6 ± 8.2	161.4 ± 8.4	161.0 ± 8.7	160.2 ± 8.9	158.8 ± 9.7	< 0.001
Body weight (kg)	65.9 ± 12.8	44.1 ± 5.4	56.8 ± 7.1	66.4 ± 7.3	73.6 ± 8.2	81.9 ± 9.6	96.6 ± 14.8	< 0.001
BMI (kg/m^2^)	25.4 ± 4.2	17.2 ± 1.1	21.9 ± 1.4	25.4 ± 0.9	28.3 ± 0.8	31.8 ± 1.3	38.3 ± 6.1	< 0.001
Hypertension	42,088 (65.4)	946 (50.8)	13,244 (57.8)	13,079 (66.3)	8634 (72.7)	4939 (76.5)	1246 (83.1)	< 0.001
Hyperlipidemia	30,339 (47.2)	541 (29.1)	9795 (42.7)	9640 (48.9)	6075 (51.2)	3389 (52.5)	789 (66.7)	< 0.001
Gout	5831 (9.1)	93 (5.0)	1539 (6.7)	1861 (9.4)	1312 (11.0)	833 (12.9)	193 (12.9)	< 0.001
History of heart failure	61 (0.1)	5 (0.3)	19 (0.1)	16 (0.1)	9 (0.1)	11 (0.2)	1 (0.1)	0.049
VHD	569 (0.9)	22 (1.2)	212 (0.9)	182 (0.9)	100 (0.8)	45 (0.7)	8 (0.5)	0.182
COPD	4076 (6.3)	210 (11.3)	1406 (6.1)	1157 (5.9)	746 (6.3)	443 (6.9)	114 (7.6)	< 0.001
CAD	4163 (6.4)	64 (3.4)	1080 (4.7)	1360 (6.9)	977 (8.2)	555 (8.6)	127 (8.5)	< 0.001
CKD	8460 (13.1)	212 (11.4)	2950 (12.8)	2598 (13.2)	1599 (13.5)	887 (13.7)	214 (14.3)	0.044
PAOD	695 (1.1)	18 (1.0)	263 (1.1)	204 (1.0)	138 (1.2)	63 (1.0)	9 (0.6)	0.288
Cancer	14,112 (21.9)	547 (29.4)	5484 (23.9)	4224 (21.4)	2434 (20.5)	1156 (17.9)	267 (17.8)	< 0.001
History of TIA/old stroke	692 (1.1)	31 (1.6)	240 (1.0)	201 (1.0)	144 (1.2)	65 (1.0)	11 (0.7)	0.054
Baseline FG (mg/dL)	136.8 ± 53.4	136.0 ± 62.9	136.8 ± 59.4	136.1 ± 49.1	136.7 ± 46.6	138.7 ± 50.8	141.2 ± 62.7	0.006
Baseline HbA1C (%)	7.2 ± 1.5	7.2 ± 1.7	7.2 ± 1.5	7.2 ± 1.4	7.3 ± 1.4	7.3 ± 1.5	7.3 ± 1.4	< 0.001
Baseline eGFR (mL/min /1.73 m^2^)	72.1 ± 32.9	64.9 ± 45.2	66.6 ± 32.2	72.0 ± 29.7	75.7 ± 31.0	82.3 ± 35.1	95.5 ± 43.0	< 0.001
CHA2DS2-VASc score	2.5 ± 1.1	2.8 ± 1.1	2.6 ± 1.1	2.5 ± 1.1	2.5 ± 1.2	2.4 ± 1.2	2.4 ± 1.1	< 0.001
UCG								
LA size (cm)	3.9 ± 0.7	3.4 ± 0.8	3.7 ± 0.7	3.9 ± 0.6	4.0 ± 0.6	4.1 ± 0.6	4.2 ± 0.7	< 0.001
DT (sec)	0.22 ± 0.07	0.21 ± 0.07	0.22 ± 0.07	0.22 ± 0.07	0.23 ± 0.07	0.22 ± 0.06	0.23 ± 0.06	< 0.001
E (mm/sec)	80.7 ± 27.4	81.7 ± 29.7	81.6 ± 28.5	79.5 ± 26.8	80.2 ± 26.4	81.8 ± 27.1	82.9 ± 26.7	< 0.001
A (mm/sec)	97.3 ± 24.9	94.5 ± 26.8	96.9 ± 26.1	97.5 ± 24.4	97.7 ± 23.7	97.6 ± 23.5	98.6 ± 25.2	0.110
E/A	0.9 ± 7.6	1.5 ± 12.2	1.1 ± 12.6	0.9 ± 2.3	0.8 ± 0.5	0.8 ± 0.4	0.8 ± 0.4	0.387
E/E’	21.5 ± 123.1	12.6 ± 6.5	20.0 ± 107.9	24.9 ± 147.7	25.6 ± 152.7	15.0 ± 54.4	11.9 ± 6.0	0.466
LVEF (%)	64.8 ± 12.4	63.3 ± 14.9	64.4 ± 13.3	65.2 ± 12.1	64.9 ± 11.8	65.3 ± 11.0	65.3 ± 10.3	< 0.001
LVIDs (cm)	3.0 ± 0.7	2.8 ± 0.8	3.0 ± 0.7	3.0 ± 0.7	3.1 ± 0.7	3.1 ± 0.6	3.2 ± 0.6	< 0.001
LVIDd (cm)	4.7 ± 0.6	4.3 ± 0.7	4.6 ± 0.7	4.8 ± 0.6	4.8 ± 0.6	4.9 ± 0.6	5.0 ± 0.6	< 0.001
LV mass (gm)	201.4 ± 61.7	158.1 ± 56.8	186.1 ± 58.8	201.4 ± 57.4	213.9 ± 60.9	225.8 ± 63.9	238.5 ± 61.1	< 0.001
Medication								
Antiplatelet	20,584 (32.0)	439 (23.6)	6524 (28.4)	6445 (32.7)	4289 (36.1)	2338 (36.2)	549 (36.6)	< 0.001
Anticoagulant	2774 (4.3)	73 (3.9)	906 (4.0)	807 (4.1)	559 (4.7)	325 (5.0)	104 (6.9)	< 0.001
CCB	27,767 (43.2)	719 (38.6)	8892 (38.8)	8531 (43.3)	5592 (47.1)	3254 (50.4)	779 (51.9)	< 0.001
Beta-blocker	19,073 (29.6)	424 (22.8)	6082 (26.5)	5873 (29.8)	3922 (33.0)	2249 (34.8)	534 (35.6)	< 0.001
ACEI/ARB	27,703 (43.1)	578 (31.0)	8570 (37.4)	8675 (44.0)	5768 (48.6)	3313 (51.3)	799 (53.3)	< 0.001
Diuretics	16,060 (25.0)	509 (27.3)	5241 (22.9)	4525 (23.0)	3202 (27.0)	2028 (31.4)	555 (37.0)	< 0.001
Statin	23,732 (36.9)	372 (20.0)	7572 (33.0)	7509 (38.1)	4937 (41.6)	2723 (42.2)	618 (41.2)	< 0.001
Metformin	32,407 (50.4)	799 (42.9)	11,174 (48.7)	10,173 (51.6)	6163 (51.9)	3344 (51.8)	754 (50.3)	< 0.001
SGLT2i	5385 (8.4)	62 (3.3)	1324 (5.8)	1641 (8.3)	1269 (10.7)	851 (13.2)	238 (15.9)	< 0.001
DDP4i	22,464 (34.9)	631 (33.9)	7793 (34.0)	6844 (34.7)	4283 (36.1)	2380 (36.9)	533 (35.5)	< 0.001
SU	21,744 (33.8)	576 (30.9)	7641 (33.3)	6667 (33.8)	4118 (34.7)	2231 (34.6)	511 (34.1)	0.010
TZD	6075 (9.4)	131 (7.0)	1956 (8.5)	1794 (9.1)	1229 (10.3)	775 (12.0)	190 (12.7)	< 0.001
Repaglinide	4712 (7.3)	206 (11.1)	1990 (8.7)	1306 (6.6)	749 (6.3)	384 (5.9)	77 (5.1)	< 0.001
Acarbose	6206 (9.6)	157 (8.4)	2180 (9.5)	1855 (9.4)	1204 (10.1)	657 (10.2)	153 (10.2)	0.054
GLP1 agonist	797 (1.2)	4 (0.2)	134 (0.6)	224 (1.1)	172 (1.4)	183 (2.8)	80 (5.3)	< 0.001
Insulin	17,769 (27.6)	828 (44.5)	6794 (29.6)	5029 (25.5)	3017 (25.4)	1691 (26.2)	410 (27.3)	< 0.001

*BMI* body mass index, *VHD* valvular heart disease, *COPD* chronic obstructive pulmonary disease, *CAD* coronary artery disease, *PAOD* peripheral arterial occlusive disease, *FPG* fasting glucose, *eGFR* estimated glomerular filtration rate, *LA* left atrium, *DT* deceleration time, *E/A* early diastolic transmitral flow velocity/late diastolic transmitral flow velocity, *E’* early diastolic mitral annular velocity, *LVEF* left ventricular ejection fraction, *LVIDd* left ventricular internal diameter in diastole, *LVIDs* left ventricular internal diameter in systole, *LV mass* left ventricle mass, *CCB* calcium channel blocker, *ACEI/ARB* angiotensin converting enzyme inhibitor/angiotensin receptor blocker, *SGLT-2 inhibitor* sodium-glucose co-transporter-2 inhibitor, *DPP4 inhibitor* dipeptidyl peptidase 4 inhibitor, *TZD* thiazolidinediones, *GLP-1 agonist* glucagon like peptide-1 agonist

The univariable and multivariable Cox regression models of the risk of AF according to BMI classification are presented in Table 2. In univariate analysis, using the normal BMI group as a reference, underweight was associated with a significantly increased risk of AF (HR 1.60, 95% CI 1.39–1.83, *p* < 0.001), while overweight was associated with a significantly reduced risk of AF (HR 0.91, 95% CI 0.85–0.97, *p* = 0.003). The risk of AF for the underweight group remained significant after multivariable adjustment for baseline risk factors and echocardiographic parameters (HR 1.52, 95% CI 1.25–1.87, *p* < 0.001). Also, the reduced risk of AF for overweight remained significant after full adjustment (HR 0.82, 95% CI 0.73–0.89, *p* < 0.001). The risks of AF for the other obesity groups (class 1 to 3) were not consistently different to that of the normal BMI group during adjustment.

**Table 2:** Adjusted hazard ratio of the risk of AF across BMI categories

BMI	Crude		Model 1		Model 2	
	HR	*P*-value	HR	*P*-value	HR	*P*-value
Continuous per unit increase	0.99 (0.98–0.99)	< 0.001	1.03 (1.02–1.03)	< 0.001	0.98 (0.97–0.99)	< 0.001
Underweight	1.60 (1.39–1.83)	< 0.001	1.33 (1.12–1.58)	0.001	1.52 (1.25–1.87)	< 0.001
Normal range	1		1		1	
Overweight	0.91 (0.85–0.97)	0.003	0.92 (0.85–0.99)	0.034	0.82 (0.73–0.89)	< 0.001
Obesity class 1	0.98 (0.91–1.06)	0.663	1.05 (0.96–1.14)	0.299	0.86 (0.78–0.95)	0.004
Obesity class 2	0.95(0.87–1.05)	0.325	1.15 (1.03–1.28)	0.014	0.85 (0.75–0.97)	0.013
Obesity class 3	1.15 (0.98–1.36)	0.084	1.50 (1.25–1.82)	< 0.001	0.89 (0.72–1.11)	0.333

Model 1 (***): adjusted for age (< 65, 65–74, ≥ 75-years-old), gender, hypertension, hyperlipidemia, gout, history of heart failure, VHD, CAD, COPD, PAOD, prior TIA/ischemic stroke, baseline HbA1C, baseline FG, CKD, and cancer

Model 2 (***): adjusted model 1, plus baseline LA size, baseline LVEF, and baseline LVM

(***): *P*-value < 0.001

**Fig. 2:**
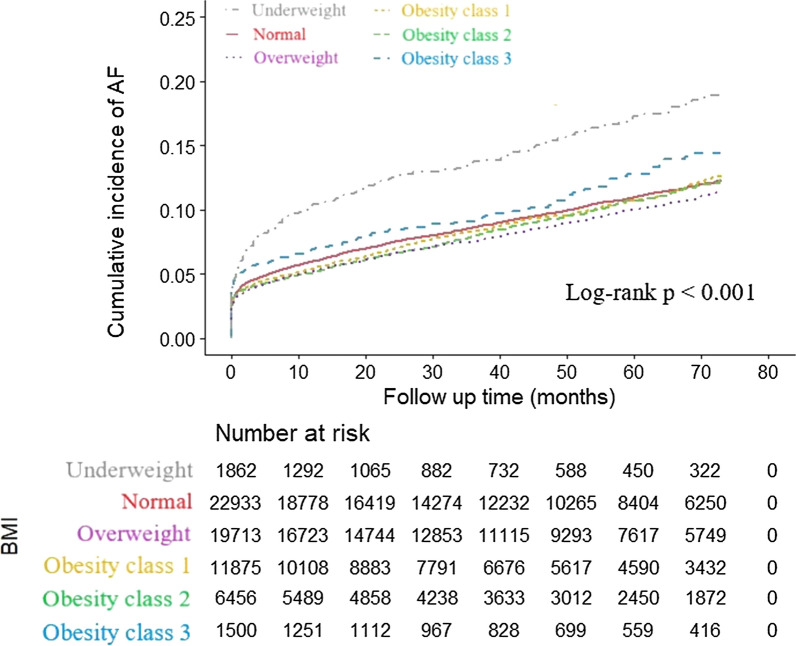
Cumulative incidence of AF for the patients with T2DM stratified by BMI categories

The curves of the cumulative incidence of AF for the different groups are illustrated in Fig. 2. The cumulative incidence of AF was highest in the underweight group, followed by the obesity class 3 group, then the normal BMI group. The overweight group had the lowest cumulative incidence of AF. The log-rank test was significant (log-rank test, *p* < 0.001).

**Fig. 3:**
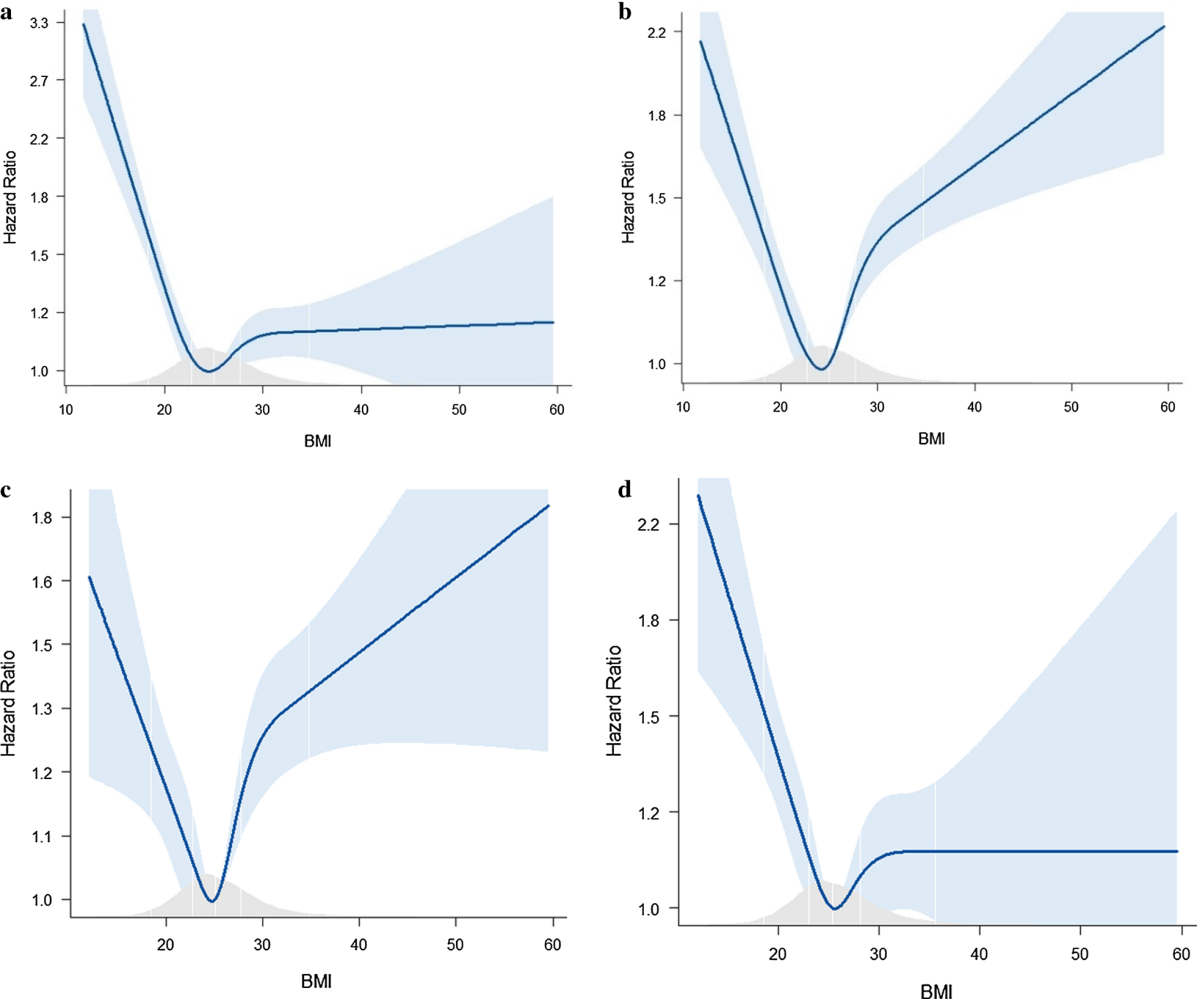
Overall and adjusted associations between BMI and AF in the crude model (**a**), model adjusted for age and gender (**b**), Model 1 (**c**), and Model 2 (**d**)

The relationship between AF risk and BMI was assessed with cubic spline models using the normal BMI group as the reference. As depicted in Fig. 3, the relationship between AF risk and BMI was initially “L-shaped” (Fig. 3a), and became “J-shaped” (Fig. 3b and c) after adjustment for age, gender, and comorbidities (model 1). After further adjustment for echocardiographic parameters, the relationship returned back to “L-shaped”, with the underweight group carrying the highest risk of AF (Fig. 3D and Additional file 1: Fig. S1).

**Fig. 4:**
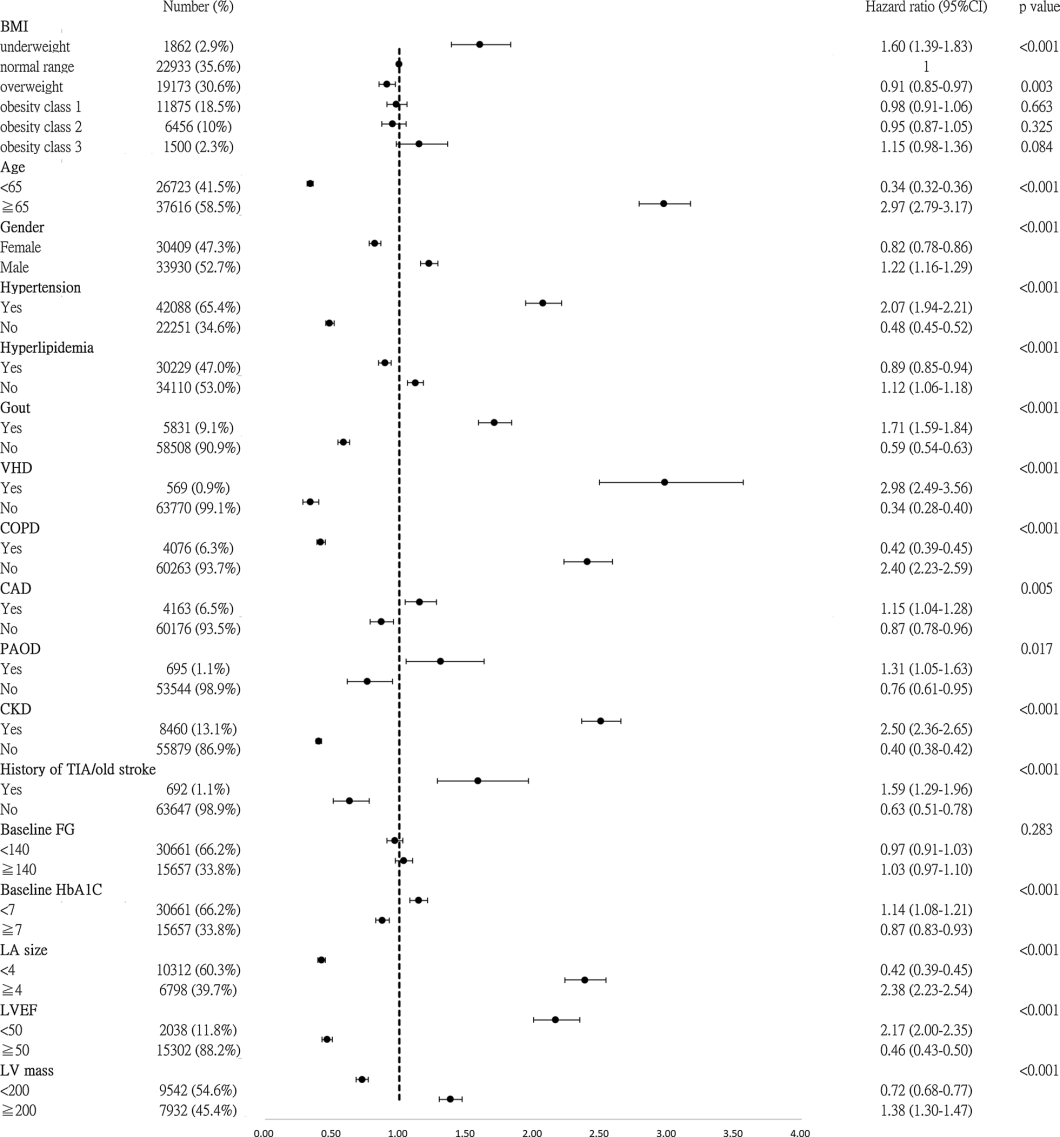
Subgroup analyses of the risk of AF

The Forest plots of the HRs in the subgroup analyses are presented in Fig. 4. Covariates including an age above 65, hypertension, gout, VHD, COPD, CAD, PAOD, CKD, history of TIA/old ischemic stroke, LA size > 4.0 cm, LVEF < 50%, and LVM more than 200 mg were associated with a higher incidence of new-onset AF.

## Discussion

Our study demonstrates a “L-shaped” relationship exists between BMI and the risk of AF in patients with T2DM. To our knowledge, this is the first dose–response analysis to observe a non-linear trend between BMI and the development of AF in Asian people with T2DM.

### The obesity paradox of AF development

There is mounting epidemiological evidence for a link between obesity and AF. The proposed mechanisms by which obesity increases the risk of AF are multifactorial, and include structural remodeling caused by increased atrial stretch, atrial fibrosis due to endothelial dysfunction, increased systemic inflammation, impaired diastolic function, enlarged LA volume, and increased deposition of pericardial fat [17, 18]. Increased LA pressure and volume and a shortened effective refractory period (ERP) in the left atrium and pulmonary vein may potentially predispose and perpetuate AF in obese patients [19]. In this study, the relationship between BMI and the risk of AF in the obese groups became insignificant after adjusting for echocardiography parameters (LA, LVEF, LVM). This suggests that the effects of obesity on AF are mainly mediated through obesity-mediated cardiac structural changes. In contrast, sustained weight loss is associated with reverse remodeling of the AF substrate and a reduction in the AF burden, in conjunction with favorable changes in the coexisting cardiometabolic risk factors [20, 21]. In an ovine model, weight loss was actually associated with structural and electrophysiological reverse remodeling and a reduced propensity for AF [22].

However, several studies have noticed that being underweight is also a risk factor for new-onset AF and poorer cardiovascular outcomes [23, 24]. In a general population, underweight was an independent risk factor for AF, only secondary to obesity class 2. One study demonstrated that a 1-unit increase in BMI was associated with a 6‒7% increased risk of AF, while a 1-unit decrease in BMI was associated with a 13% increase in AF risk [25]. Moreover, both obesity and underweight were associated with a higher rate of AF recurrence after catheter ablation [26].

### Potential pathophysiology of the obesity paradox in AF development

Data from the ORIGIN trial revealed that obesity and weight loss are inversely related to mortality and cardiovascular outcomes in patients with T2DM [27]. A recent meta-analysis also showed that an obesity paradox exists with respect to all-cause and cardiovascular mortality [28]. There are several potential mechanisms underlying the obesity paradox for heart failure and cardiovascular mortality. These include obesity-related paradoxically increased mobilization of endothelial progenitor cells, increased ghrelin sensitivity, decreased thromboxane production, and decreased TNF levels. In addition, several adipokines produced by adipose tissue have been shown to be cardiovascular-protective [29]. Moreover, activation of the sympathetic nervous system has been suggested to be less toxic in obesity-related hypertension [30]. Although the mechanisms underlying the “L-shaped” phenomenon observed in our study are unknown, it is likely that both inflammatory cytokines and the autonomic nervous system may contribute to the risk of AF. It is well-known that a normal BMI with a larger waist circumference and poorer metabolic profile is more prevalent in non-whites; this status is characterized as “metabolically unhealthy normal-weight” [31, 32].

Another possibility is variability in body weight, although data on this factor was not available in this study. Research has demonstrated that long-term changes in body weight, either loss or gain in BMI, have cumulative effects on AF risk [33]. Additionally, high bodyweight variability was independently associated with the development of AF in patients with T2DM, and this association was stronger in underweight patients and those with advanced-stage diabetes [34]. While intentional weight loss is beneficial for obese individuals, unintentional weight loss may be related to hidden chronic diseases and cachexia, which may increase the susceptibility to AF.

### Optimal body weight for Asian people with DM

Debate continues regarding the existence of an obesity paradox. One major issue is the failure to address confounding variables and reverse causation biases in studies. For example, detailed data on potential mediator lifestyle factors—such as smoking, alcohol drinking, chronic diseases such as cancer, or low physical activity—needs to be collected and properly analyzed [35]. This is particularly true for alcohol intake, as consumption of alcohol has been demonstrated to increase the risk of incident AF [36, 37]. Another concern is the utility of BMI to accurately reflect nutritional status, such as body composition and body fat distribution [38]. Evidence has shown that abdominal obesity may more accurately predict AF in nonobese Asians than BMI [39]. Nevertheless, BMI is still considered as important as or even more important than the total adiposity measures, which can only be assessed using complex and expensive methods, and BMI has been consistently proven to be a strong predictor of cardiovascular outcomes [40].

The WHO has proposed lower BMI cutoff values for defining overweight (BMI ≥ 23 kg/m^2^) and obesity (BMI ≥ 25 kg/m^2^) in Asian populations, though most of this evidence comes from cross-sectional studies. In line with previous studies that do not support the use of lower BMI cutoff values for overweight and obesity in Asian populations [41], our study adopted the BMI criteria suggested by the government of Taiwan. Furthermore, in our study, underweight (BMI < 18.5) was associated with the highest risk for AF, and the risk of AF was even higher for this group than the obesity class 3 group (BMI ≥ 35) in the fully adjusted model. Obesity only represents one potential target for lifestyle modifications to reduce the occurrence of AF, thus the results of our study should be interpreted with caution and confirmed by further studies.

## Limitations

This study has some limitations. First, since we did not measure body weight at every outpatient clinic visit, the influence of temporal changes of BMI could not be addressed. Second, other obesity-related parameters such as waist circumference or physical activity were not assessed in this study. Third, data on other potential confounders, such as smoking status, alcohol intake, socioeconomic status, physical activity, and obstructive sleep apnea, were not available in our database. Inflammatory biomarkers such as C-reactive protein (hsCRP) are also strong confounding factors. However, hsCRP was not routinely recorded in our database and was only measured to monitor acute inflammation or infection. Fourth, we excluded subjects who were not consistently followed at our hospital, since some outcome data may be missing for these patients. This approach may have led to selection bias, but ensured that all the outcomes were accurately determined. Finally, our conclusions are based on a retrospective single-center analysis, and further studies are warranted to confirm whether these results can be generalized to other populations.

## Conclusion

Underweight is a major risk factor for the development of AF among patients with T2DM.

## Supplementary Information

**Additional file 1.** Supplementary data. **Table S1.** ICD codes. **Table S2.** Number of visits during follow up in each subgroup. **Figure S1.** Hazard ratios for developing AF across different BMI categories separated by gender.

## Data Availability

The datasets used in this study are only available at National Taiwan University Hospital. The R programs (codes) used in this study are available from the corresponding author on reasonable request.

## Abbreviations

ACEI: Angiotensin converting enzyme inhibitor
AF: Atrial fibrillation
ARB: Angiotensin receptor blocker
BMI: Body mass index
HTN: Hypertension
CAD: Coronary artery disease
CCB: Calcium channel blocker
CKD: Chronic kidney disease
COPD: Chronic obstructive pulmonary disease
DOAC: Direct oral anticoagulant
DPP4: Dipeptidyl peptidase 4
eGFR: Estimated glomerular filtration rate
EHRs: Electronic health records
GLP-1: Glucagon like peptide-1
LA: Left atrium
LVEF: Left ventricular ejection function
LVIDd: Left ventricular internal dimension in end‐diastole
LVIDs: Left ventricular internal dimension in systole
LVM: Left ventricular mass
MDRD: Modification of diet in renal disease
MRA: Mineralocorticoid-receptor antagonist
PAOD: Peripheral arterial occlusive disease
SGLT2: Sodium-glucose co-transporter-2
T2DM: Type 2 diabetes mellitus
TIA: Transient ischemic accident
TZD: Thiazolidinedione
VHD: Valvular heart disease

## References

[1] Staszewsky L, Cortesi L, Baviera M, Tettamanti M, Marzona I, Nobili A (2015). Diabetes mellitus as risk factor for atrial fibrillation hospitalization: Incidence and outcomes over nine years in a region of Northern Italy. Diabetes Res Clin Pract.

[2] Béland MJ, Harris KC, Marelli AJ, Houyel L, Bailliard F, Dallaire F (2018). Improving quality of congenital heart disease research in Canada: standardizing nomenclature across Canada. Can J Cardiol..

[3] Pallisgaard JL, Schjerning AM, Lindhardt TB, Procida K, Hansen ML, Torp-Pedersen C, Gislason GH (2016). Risk of atrial fibrillation in diabetes mellitus: a nationwide cohort study. Eur J Prev Cardiol..

[4] Menke A, Casagrande S, Geiss L, Cowie CC (2015). Prevalence of and trends in diabetes among adults in the United States, 1988–2012. JAMA..

[5] Bhupathiraju SN, Hu FB (2016). Epidemiology of obesity and diabetes and their cardiovascular complications. Circ Res..

[6] Wanahita N, Messerli FH, Bangalore S, Gami AS (2008). Atrial fibrillation and obesity—results of a meta-analysis. Am Heart J..

[7] Tedrow UB, Conen D, Ridker PM, Cook NR, Koplan BA, Manson JE, Buring JE, Albert CM (2010). The long- and short-term impact of elevated body mass index on the risk of new atrial fibrillation the WHS (women's health study). J Am Coll Cardiol..

[8] Wang TJ, Parise H, Levy D (2004). Obesity and the risk of new-onset atrial fibrillation. JAMA..

[9] Berkovitch A, Kivity S, Klempfner R, Segev S, Milwidsky A, Erez A (2016). Body mass index and the risk of new-onset atrial fibrillation in middle-aged adults. Am Heart J.

[10] Kim YG, Han KD, Choi JI, Boo KY, Kim DY, Oh SK (2019). The impact of body weight and diabetes on new-onset atrial fibrillation: a nationwide population based study. Cardiovasc Diabetol..

[11] Lee JW, Brancati FL, Yeh HC (2011). Trends in the prevalence of type 2 diabetes in asians versus whites: results from the united states national health interview survey, 1997–2008. Diabetes Care.

[12] Gujral UP, Mohan V, Pradeepa R, Deepa M (2018). Ethnic differences in the prevalence of diabetes in underweight and normal weight individuals: The CARRS and NHANES studies. Diabetes Res Clin Pract..

[13] Barakat AF, Jain S, Masri A, Alkukhun L, Senussi M, Sezer A (2021). Outcomes of direct oral anticoagulants in atrial fibrillation patients across different body mass index categories. JACC Clin Electrophysiol..

[14] Park CS, Choi EK, Kim HM, Lee SR, Cha MJ (2017). Increased risk of major bleeding in underweight patients with atrial fibrillation who were prescribed non-vitamin K antagonist oral anticoagulants. Heart Rhythm..

[15] Grymonprez M, Capiau A, De Backer TL, Steurbaut S (2021). The impact of underweight and obesity on outcomes in anticoagulated patients with atrial fibrillation: a systematic review and meta-analysis on the obesity paradox. Clin Cardiol..

[16] Desquilbet L, Mariotti F (2010). Dose-response analyses using restricted cubic spline functions in public health research. Stat Med.

[17] Nalliah CJ, Sanders P, Kottkamp H, Kalman JM (2016). The role of obesity in atrial fibrillation. Eur Heart J..

[18] Kosiuk J, Van Belle Y, Bode K, Kornej J, Arya A, Rolf S (2012). Left ventricular diastolic dysfunction in atrial fibrillation: predictors and relation with symptom severity. J Cardiovasc Electrophysiol.

[19] Munger TM, Dong YX, Masaki M, Oh JK, Mankad SV (2012). Electrophysiological and hemodynamic characteristics associated with obesity in patients with atrial fibrillation. J Am Coll Cardiol..

[20] Abed HS, Wittert GA, Leong DP, Shirazi MG, Bahrami B, Middeldorp ME (2013). Effect of weight reduction and cardiometabolic risk factor management on symptom burden and severity in patients with atrial fibrillation: a randomized clinical trial. JAMA..

[21] Pathak RK, Middeldorp ME, Meredith M, Mehta AB, Mahajan R (2015). Long-term effect of goal-directed weight management in an atrial fibrillation cohort: a long-term follow-up study (LEGACY). J Am Coll Cardiol..

[22] Mahajan R, Lau DH, Brooks AG, Shipp NJ, Wood JP, Manavis J (2021). Atrial fibrillation and obesity: reverse remodeling of atrial substrate with weight reduction. JACC Clin Electrophysiol..

[23] Elagizi A, Kachur S, Lavie CJ, Carbone S, Pandey A, Ortega FB (2018). An overview and update on obesity and the obesity paradox in cardiovascular diseases. Prog Cardiovasc Dis..

[24] Bhaskaran K, Santos-Silva I, Leon DA, Douglas IJ (2018). Association of BMI with overall and cause-specific mortality: a population-based cohort study of 3.6 million adults in the UK. Lancet Diabetes Endocrinol..

[25] Kang S-H, Choi E-K, Han K-D, Lee S-R, Lim W-H, Cha M-J (2016). Underweight is a risk factor for atrial fibrillation: A nationwide population-based study. Int J Cardiol.

[26] Deng H, Shantsila A, Guo P, Potpara TS, Zhan X (2018). A U-shaped relationship of body mass index on atrial fibrillation recurrence post ablation: a report from the Guangzhou atrial fibrillation ablation registry. EBioMedicine..

[27] Deng H, Shantsila A, Guo P, Potpara TS, Zhan X (2020). Obesity and weight loss are inversely related to mortality and cardiovascular outcome in prediabetes and type 2 diabetes: data from the ORIGIN trial. Eur Heart J..

[28] Kwon Y, Kim HJ, Park S, Park YG, Cho KH (2017). Body mass index-related mortality in patients with type 2 diabetes and heterogeneity in obesity paradox studies: a dose-response meta-analysis. PLoS ONE..

[29] Hainer V, Aldhoon-Hainerová I (2013). Obesity paradox does exist. Diabetes Care..

[30] Esler M, Lambert G, Schlaich M, Dixon J, Sari CI, Lambert E (2018). Obesity paradox in hypertension: is this because sympathetic activation in obesity-hypertension takes a benign form?. Hypertension..

[31] Eckel N, Mühlenbruch K, Meidtner K, Boeing H (2015). Characterization of metabolically unhealthy normal-weight individuals: Risk factors and their associations with type 2 diabetes. Metabolism..

[32] Gujral UP, Narayan KM (2019). Diabetes in normal-weight individuals: high susceptibility in nonwhite populations. Diabetes Care..

[33] Feng T, Vegard M, Strand LB, Laugsand LE (2019). Weight and weight change and risk of atrial fibrillation: the HUNT study. Eur Heart J..

[34] Lee HJ, Choi EK, Han KD, Kim DH, Lee E (2020). High variability in bodyweight is associated with an increased risk of atrial fibrillation in patients with type 2 diabetes mellitus: a nationwide cohort study. Cardiovasc Diabetol..

[35] Preston SH, Stokes A (2014). Obesity paradox conditioning on disease enhances biases in estimating the mortality risks of obesity. Epidemiology.

[36] Csengeri D, Sprünker NA, Di Castelnuovo A, Niiranen T, Vishram-Nielsen JK (2021). Alcohol consumption, cardiac biomarkers, and risk of atrial fibrillation and adverse outcomes. Eur Heart J..

[37] Tu SJ, Gallagher C, Elliott AD, Linz D, Pitman BM, et al. Risk thresholds for total and beverage-specific alcohol consumption and incident atrial fibrillation. JACC Clin Electrophysiol. 2021;S2405–500X(21)00524–7. 10.1016/j.jacep.2021.05.013 34330672

[38] Carbone S, Lavie CJ (2019). An opposing point of view on the obesity paradox. Postgrad Med..

[39] Baek YS, Yang PS, Kim TH, Uhm JS, Park J, Pak HN (2017). Associations of abdominal obesity and new-onset atrial fibrillation in the general population. J Am Heart Assoc..

[40] Ortega FB, Sui X, Lavie CJ, Blair SN (2016). Body mass index, the most widely used but also widely criticized index: would a criterion standard measure of total body fat be a better predictor of cardiovascular disease mortality?. Mayo Clin Proc..

[41] Lin WY, Tsai SL, Albu JB, Lin CC, Li TC, Pi-Sunyer FX (2011). Body mass index and all-cause mortality in a large Chinese cohort. CMAJ..

