# Supplementary material for: Underweight is a major risk factor for atrial fibrillation in Asian people with type 2 diabetes mellitus
Source: Cardiovasc Diabetol. 2021 Nov 24;20:226. doi: 10.1186/s12933-021-01415-2 (PMC8613951; doi:10.1186/s12933-021-01415-2)
Supplement: Supplementary file 1 — Additional file 1. Supplementary data. Table S1. ICD codes. Table S2. Number of visits during follow up in each subgroup. Figure S1. Hazard ratios for developing AF across different BMI categories separated by gender. [file 12933_2021_1415_MOESM1_ESM.docx]

**Table S1. ICD codes**

| Diagnosis | ICD 9 | ICD 10 |
| --- | --- | --- |
| Hypertension | 401.0, 401.1, 401.9, 403.00, 403.01, 403.10, 403.11, 409.90, 403.91, 404.01, 404.91, 405.99 | I10, I11.0, I11.9, I12.0, I13.0, I15.9 |
| Hyperlipidemia | 272.0, 272.1, 272.3, 272.4, 272.8 | E75.5, E78.0, E78.1, E78.2, E78.3, E78.4, E78.5, E78.9 |
| Gout | 274.0, 274.11, 274.82, 274.89 | M10.00, M10.9, M1A.9X, M10.011, M10.012, M10.019, M10.021, M10.022, M10.029, M10.031, M10.032, M10.039, M10.041, M10.042, M10.049, M10.051, M10.059, M10.061, M10.062, M10.069, M10.071  N20.0, M10.40, M10.411, M10.412, M10.419, M10.421, M10.422, M10.429, M10.431  M10.432, M10.439, M10.441, M10.442, M10.449, M10.451  M10.452, M10.459, M10.461, M10.462, M10.469, M10.71 |
| Chronic obstructive pulmonary disease | 491.20, 491.21, 491.8, 492.8, 493.20, 493.22, 493.90, 493.91, 494.0, 494.1, 496 | J44.9, J44.0, J44.1, J41.8, J44.9, J45.902, J45.991, J45.909, J45.991, J45.998, J47.9, J47.0, J47.1, J44.9 |
| Heart failure | 402.11, 402.91, 404.11, 428.0, 428.1, 428.9 | I50.1, I50.20, I50.23, I50.31, I50.9 |
| Peripheral arterial occlusive disease | 443.81, 443.89, 443.9, 444.0, 444.21, 444.22, 444.81, 444.9 | I73.89, I73.9, I74.09, I74.19  I74.2, I74.3, I74.4, I74.9 |
| Atrial fibrillation | 427.31 | I48.0, I48.1, I48.2, I48.91 |
| Atrial flutter | 427.32 | I48.4, I48.92 |
| Transient ischemia accident | 435.9 | G45.3, G45.4, G45.8, G45.9 |
| Ischemic stroke | 436 | I67.89 |
| Coronary artery disease | 411.81, 414.00, 414.01, 414.02, 414.03, 414.04, 414.8, 414.9 | I20.0, I20.9, I25.10, I25.11, I25.82, I25.84, I25.9 |
| Acute myocardial infarction | 410.00, 410.01, 410.02, 410.10, 140.11, 410.12, 410.20, 410.21, 410.22, 410.30, 410.31, 410.32, 410.40, 410.41, 410.42, 410.50, 410.51, 410.52, 410.61, 410.81 | I21.01, I21.02, I21.09, I21.11, I21.19, I21.21, I21.29, I21.3, I21.4, I22.1, I22.8, I24.9 |
| Valvular heart disease | 394.0, 394.1, 394.2, 394.9, 395.0, 395.1, 395.2, 395.9, 396.0, 396.1, 396.2, 396.3, 396.8, 396.9, 397.0, 397.1, 397.9, 398.0, 398.90, 398.91, 398.99., 424.0, 424.1, 424.2, 424.3, 424.90, 424.91, 424.99 | I05.0, I05.1, I05.2, I05.8, I05.9, I06.0, I06.1, I06.2, I06.8, I06.9, I07.0, I07.1, I07.2, I07.8, I07.9, I08.0, I08.1, I08.2, I08.3, I08.8, I08.9, I09.0, I09.1, I09.2, I09.81, I09.89, I09.9, I34.0, I34.1, I34.2, I34.8, I34.9, I35.0, I35.1, I35.2, I35.8, I35.9, I36.0, I36.1, I36.2, I36.8, I36.9, I37.0, I37.1, I37.2, I37.8, I37.9 |
| Chronic kidney disease | 593.9 | N18.1, N18.2, N18.3, N18.4, N18.5, N18.9, N19 |
| Cancer | ICD - O International Classification of Diseases for Oncology  C00~C80 | |

**Table S2. Number of visits during follow up in each subgroup**

|  | Underweight | Normal | Overweight | Obesity class 1 | Obesity class 2 | Obesity class 3 |
| --- | --- | --- | --- | --- | --- | --- |
| Outpatient visits (mean, Q1~Q3) | 37.8 (6.0-49.5) | 43.2 (8.0-60.0) | 43.1 (9.0-60.0) | 42.9 (9.0-60.0) | 42.6 (8.0-59.0) | 42.8 (8.0-60.0) |
| Inpatient visits (mean, Q1~Q3) | 2.6 (1.0-3.0) | 1.0 (1.0-3.0) | 1.0 (1.0-3.0) | 2.6 (1.0-3.0) | 1.0 (1.0-3.0) | 1.0 (1.0-3.0) |
| BMI (mean, Q1~Q3) | 31.9 (7.0-45.0) | 33.9 (6.0-47.0) | 32.3 (6.0-45.0) | 32.5 (6.0-45.0) | 32.8 (6.0-45.0) | 32.9 (6.0-45.0) |

**Figure S1. Hazard ratios for developing AF across different BMI categories separated by gender**

| Male | Female |
| --- | --- |
| 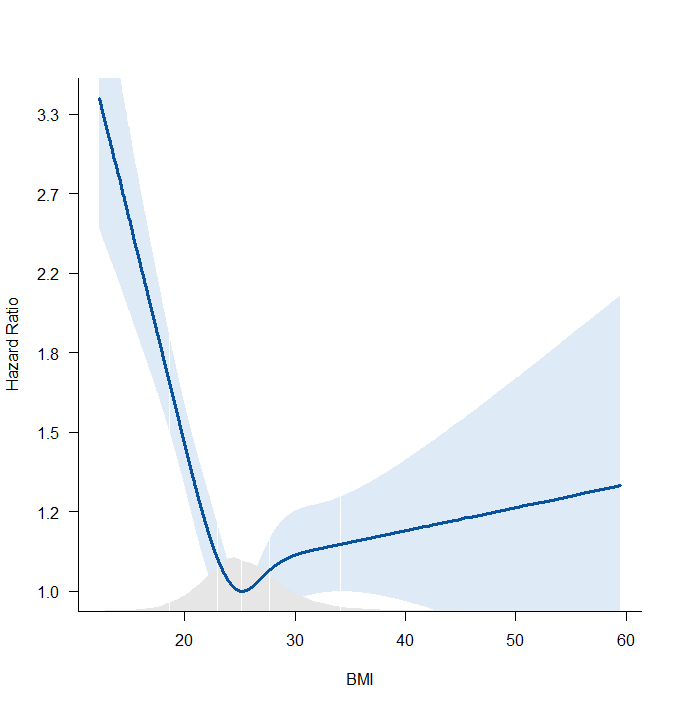 | 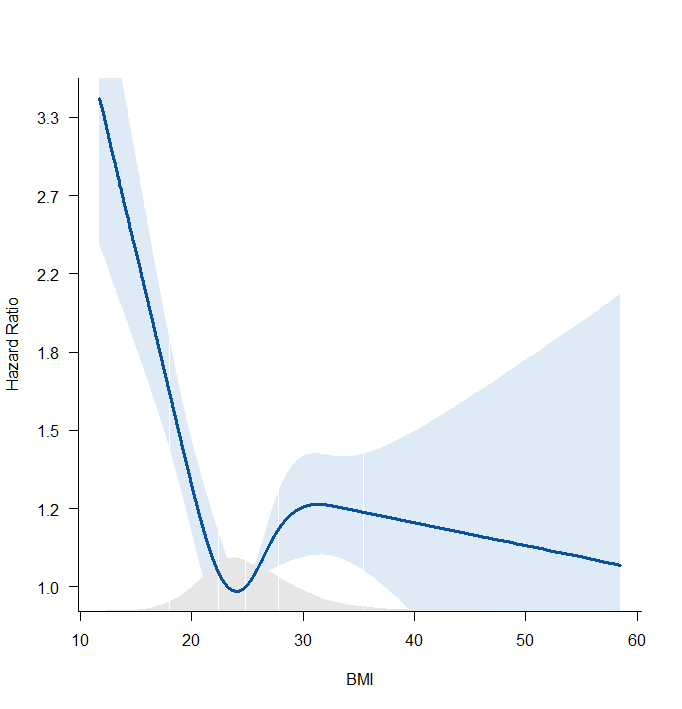 |
| Age adjusted (Male) | Age adjusted (Female) |
| 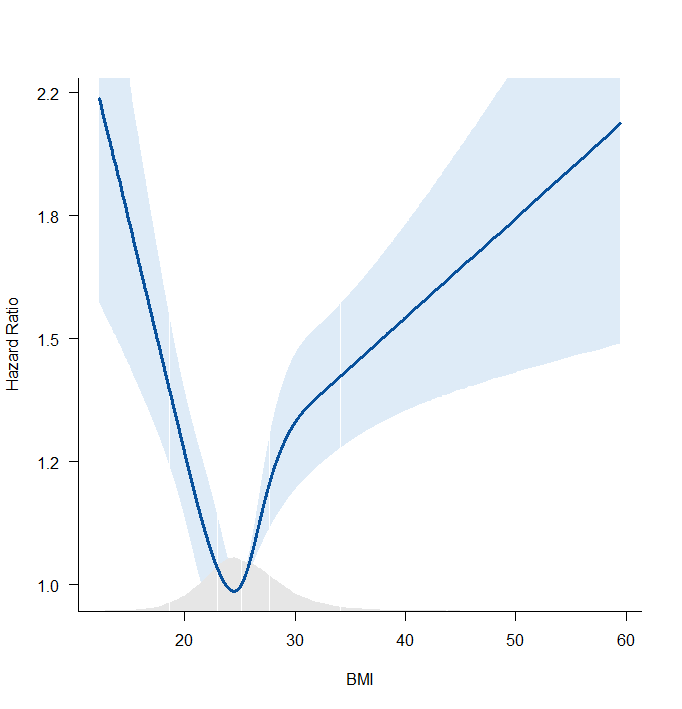 | 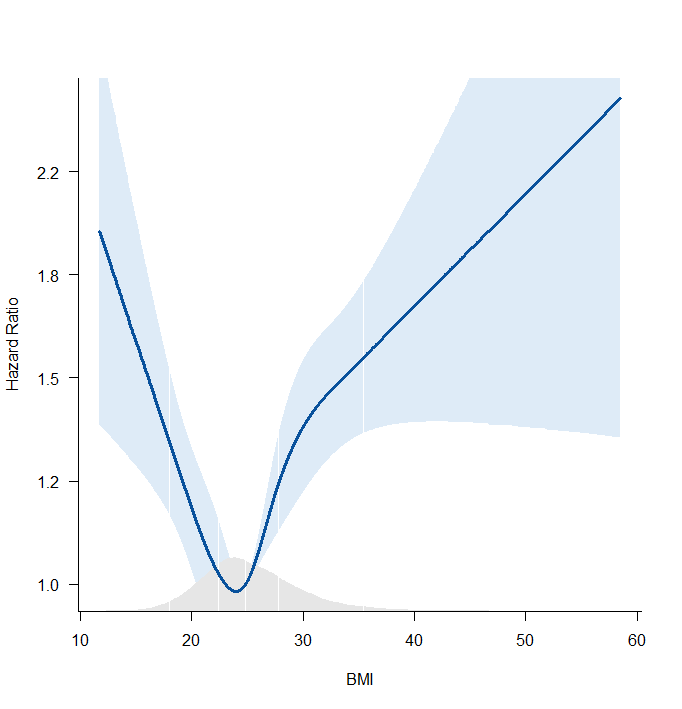 |
| Model 1 (Male) | Model 1 (Female) |
| 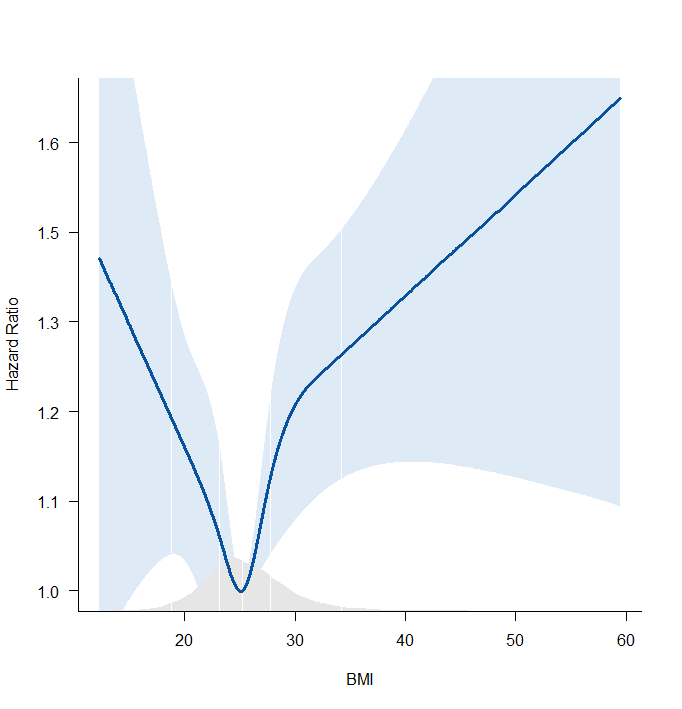 | 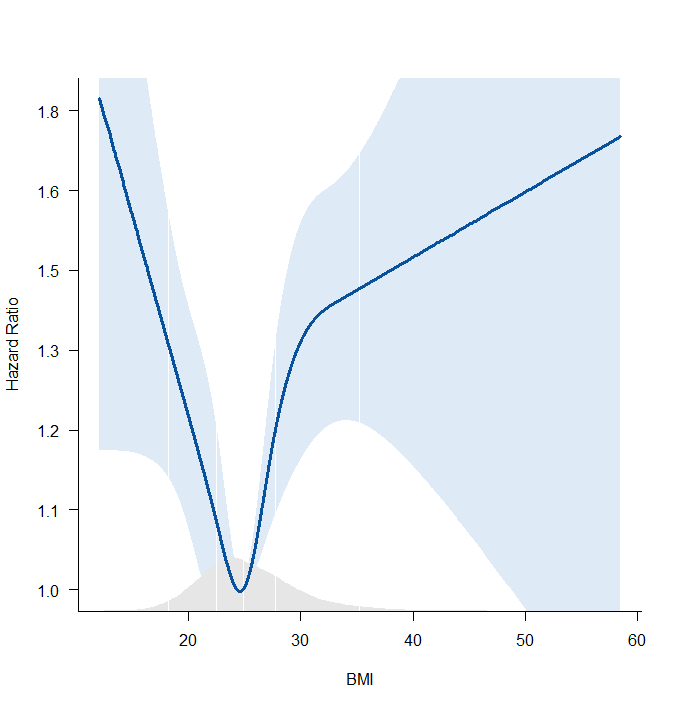 |
| Model 2 (Male) | Model 2 (Female) |
| 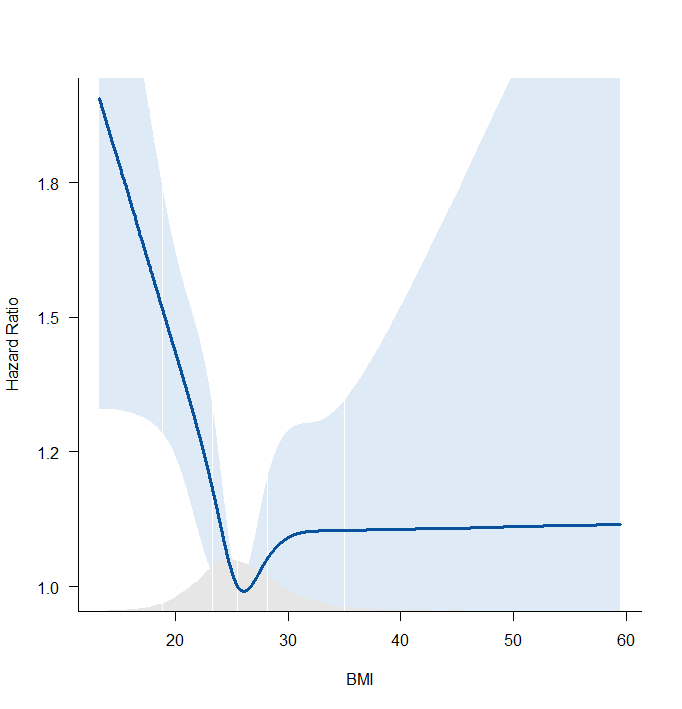 | 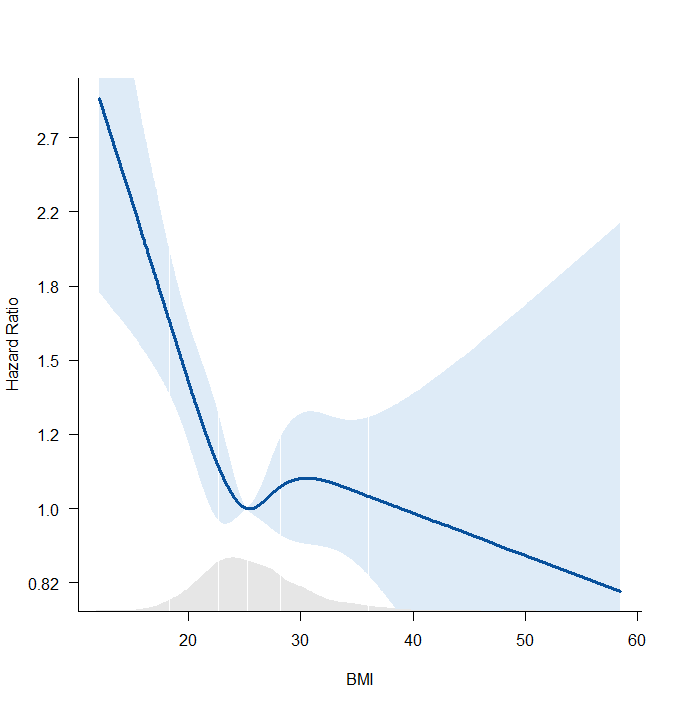 |
